# Markov Jump Linear Systems-Based Position Estimation for Lower Limb Exoskeletons

**DOI:** 10.3390/s140101835

**Published:** 2014-01-22

**Authors:** Samuel L. Nogueira, Adriano A. G. Siqueira, Roberto S. Inoue, Marco H. Terra

**Affiliations:** 1 Department of Mechanical Engineering, University of São Paulo, São Carlos, SP 13566-590, Brazil; E-Mail: samlourenco@gmail.com; 2 Center for Robotics of São Carlos and Center for Advanced Studies in Rehabilitation, University of São Paulo, SP 13566-590, Brazil; E-Mail: terra@sc.usp.br; 3 Department of Electrical Engineering, Federal University of São Carlos, SP 13565-905, Brazil; E-Mail: rsinoue@ufscar.br; 4 Department of Electrical Engineering, University of São Paulo, São Carlos, SP 13566-590, Brazil

**Keywords:** discrete-time systems, Markovian jump, Kalman filter, ortheses, exoskeleton, wearable robots, lower limbs

## Abstract

In this paper, we deal with Markov Jump Linear Systems-based filtering applied to robotic rehabilitation. The angular positions of an impedance-controlled exoskeleton, designed to help stroke and spinal cord injured patients during walking rehabilitation, are estimated. Standard position estimate approaches adopt Kalman filters (KF) to improve the performance of inertial measurement units (IMUs) based on individual link configurations. Consequently, for a multi-body system, like a lower limb exoskeleton, the inertial measurements of one link (e.g., the shank) are not taken into account in other link position estimation (e.g., the foot). In this paper, we propose a collective modeling of all inertial sensors attached to the exoskeleton, combining them in a Markovian estimation model in order to get the best information from each sensor. In order to demonstrate the effectiveness of our approach, simulation results regarding a set of human footsteps, with four IMUs and three encoders attached to the lower limb exoskeleton, are presented. A comparative study between the Markovian estimation system and the standard one is performed considering a wide range of parametric uncertainties.

## 1. Introduction

Research and development on robotic devices for rehabilitation and assistance have received much attention in the last few years. Alternative designs of exoskeletons for upper and lower limbs have been proposed in the literature (e.g., [1–3]). We can cite force and impedance controls as examples to suit the patient's gait pattern and to provide new approaches for enhancing balance and muscle strengthening.

As the control approaches for this class of robots are generally performed based on absolute and relative angles of their segments, they depend on reliable and accurate position measurements. In this sense, inertial measurement units (IMUs) have been successfully applied to gait identification (e.g., [4,5]) and in control approaches to provide stability and gait-pattern adaptation during walking. In [6], for instance, the use of an IMU is proposed to estimate the knee joint angle of a lower limb orthosis. The resulting measurement provides redundant information to the control system, conceived to dynamically adapt the impedances of the orthosis joints.

In [7], switch sensors based on force sensitive resistors (FSR) and IMUs are used to determine the phases of the human gait, namely heel-strike, toe-off, stance and swing phases. The authors use gait-phase identification to improve the accelerometer performance. However, it does not provide a good indication of accelerometer reliability of all lower limb segments. Since the dynamics are different for each segment during walking, this approach provides a good result only for the foot segment. The acceleration measurements deteriorate when we take into account other members of lower limbs.

In order to deal with the performance limitations of lower limb exoskeletons, in [8], three accelerometers are used instead of identifying gait phases, in an approach based on Kalman filter and genetic algorithm optimization. A robust estimation version of this approach is proposed in [9], whose effectiveness is verified in extreme conditions of parametric uncertainties.

It is known that when the dynamic acceleration increases, the number of reliable accelerometers reduces at a certain instant of time. Consequently, in some segments, like the trunk and foot, the high incidence of dynamic accelerations can degrade the angular estimation. Furthermore, human walking has great variability and unpredictability related with the speed and length of each step, which cause different patterns of dynamic accelerations in each segment. In order to deal with this problem, we propose a model that encompasses the influence of whole dynamic accelerations in a lower limb exoskeleton.

This model is developed based on Markovian systems, which are a particular class of randomly time varying-systems. The main feature of this approach is that the probability of the system being in a different configuration in the next time instant depends only on the present system configuration, regardless of the time instant considered. Markovian modeling has been used to solve control problems in robotic systems. For example, in [10], a position Markovian 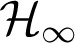
_∞_ controller was implemented in an underactuated manipulator, subject to abrupt changes on its configuration. It is conceived of to deal with a set of joint faults.

In this paper, we propose a new approach based on the Markovian model to estimate angular positions in lower limbs exoskeleton to overcome the accelerometer reliability problem over all gait phases. In contrast to [4–9], where the position estimations are based on an individual link configuration, the proposed method takes into account a collective modeling of all inertial sensors attached to the exoskeleton.

In order to show the effectiveness of the estimation model proposed, we present simulations based on a set of human footsteps, with four IMUs and three encoders attached to the lower limb exoskeleton. A comparative study between estimations based on Markovian and standard Kalman filters is performed, considering a wide range of parametric uncertainties.

This paper is organized as follows: Section 2 presents the accelerometer and gyroscope models, the standard sensor fusion strategy and the proposed Markov Jump Linear Systems (MJLS)-based model. Section 3 presents the Markovian Kalman filter algorithm and the required conditions to select the best information from each sensor. Finally, Section 4 presents simulated results and discussions.

## 2. Markovian Estimation Model

**Figure 1.**
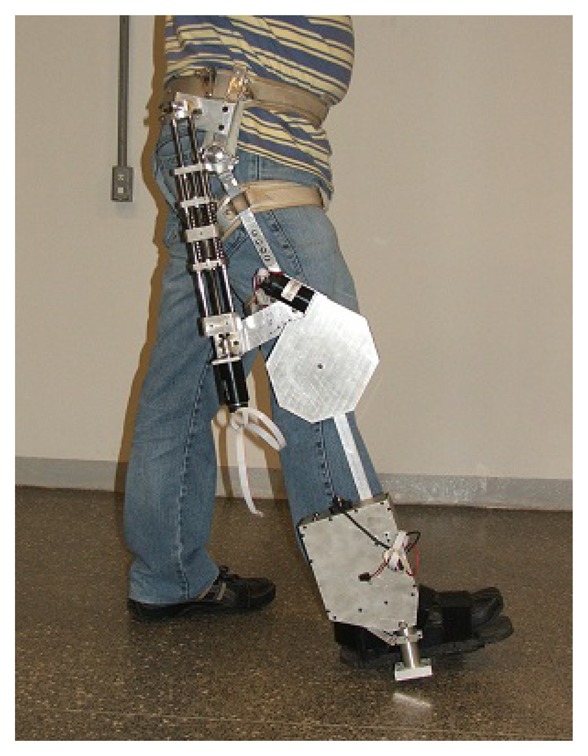
The end of the swing phase of a step through the Exo-Kanguera.

In this section, the Markovian model and the sensor fusion strategy designed to estimate the absolute and relative angular positions of exoskeletons for lower limbs are presented. The proposed approach will be applied to the exoskeleton, Exo-Kanguera, where rehabilitation protocols have been used to help stroke and spinal cord injured patients during walking rehabilitation. It is driven by a series of elastic actuators that allow the implementation of impedance control, ensuring patient safety and the ability to define different interaction behaviors. Figure 1 shows a user with the Exo-Kanguera.

In the following, gyroscope and accelerometer models are described. Standard sensor fusion approaches for an individual IMU and the proposed MJLS-based model, which includes signals of all IMUs attached to the exoskeleton, are presented.

### 2.1. Gyroscope and Accelerometer Models

**Figure 2.**
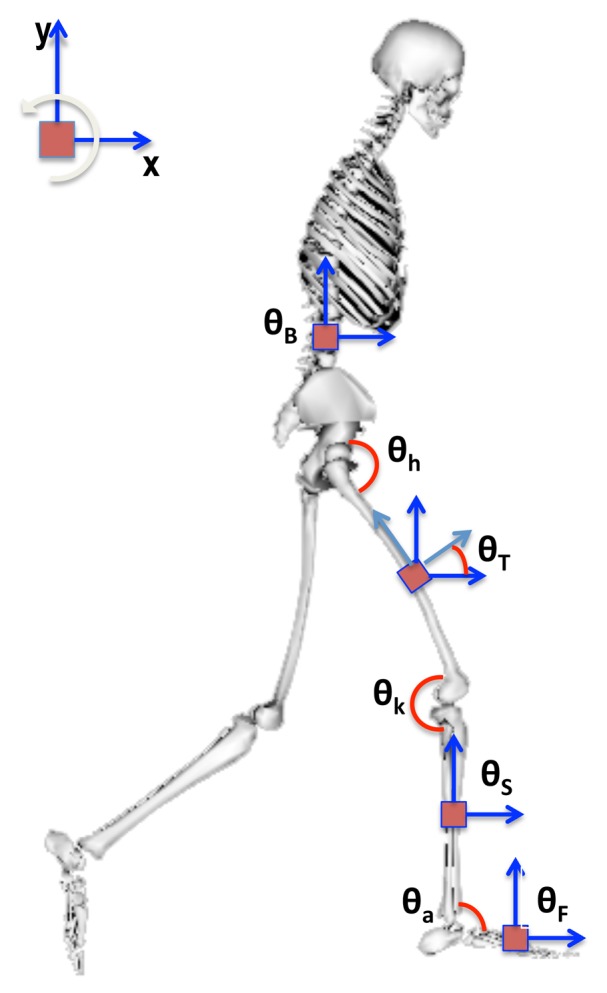
Inertial measurement units (IMUs) attached to the body

The IMUs are attached to each segment of the body, as shown in Figure 2. Regarding the gyroscope measurement, the angular velocity of the link where the IMU is attached, *θ̇*(*t*), at a given instant, *t*, is modeled as:

(1) $$\dot{\theta }(t)={\dot{\theta }}_{\text{g}}(t)+b_{\text{g}}(t)+\eta_{\text{g}}(t)$$

(2) $${\dot{b}}_{\text{g}}(t)=-\frac{1}{\tau_{\text{g}}}b_{\text{g}}(t)+\eta_{b_{\text{g}}}(t)$$

where *θ̇*_g_(*t*) is the angular velocity measured from the gyroscope in the *z*-axis, *η*_g_(*t*) is white Gaussian noise with variance $\sigma_{\text{g}}^{2}$, *b*_g_(*t*) is the gyroscope bias, *τ*_g_ is the Markov process correlation time and *η_b_*_g_(*t*) is white Gaussian noise with variance $\sigma_{b_{\text{g}}}^{2}$.

Analogously, the acceleration of the link, *a*(*t*), at a given instant, *t*, is modeled as:

(3) $$a(t)=a_{\text{a}}(t)+b_{\text{a}}(t)+\mu_{\text{a}}(t)$$

(4) $${\dot{b}}_{\text{a}}(t)=-\frac{1}{\tau_{\text{a}}}b_{\text{a}}(t)+\mu_{b_{\text{a}}}(t)$$

where *a*_a_(*t*) is the acceleration obtained from the accelerometer in the *x*-axis of the local coordinate system, *μ*_a_(*t*) is white Gaussian noise with variance $\sigma_{\text{g}}^{2}$, *b*_a_(*t*) is the accelerometer bias, *τ*_a_ is the Markov process correlation time and *μ_b_*_a_(*t*) is white Gaussian noise with variance $\sigma_{b_{\text{a}}}^{2}$.

Since the variances, $\sigma_{\text{g}}^{2}$, $\sigma_{b_{\text{g}}}^{2}$, $\sigma_{\text{a}}^{2}$ and $\sigma_{b_{\text{a}}}^{2}$, are known, simulated signals for *θ̇*_g_ and *a*_a_ can be generated considering a set of curves for *θ̇* and *a*, defined from normal gait patterns.

### 2.2. Sensor Fusion Strategy

Standard sensor fusions based on Kalman filters take into account the gyroscope signal as the main source for position estimation. They use the accelerometer signal as a redundant information to correct the estimated value from the gyroscope, usually corrupted by integration errors. The estimated angular velocity and bias from the gyroscope are given by:

(5) $${\dot{\hat{\theta }}}_{\text{g}}(t)={\dot{\theta }}_{\text{g}}(t)+{\hat{b}}_{\text{g}}(t)$$

(6) $${\dot{\hat{b}}}_{\text{g}}(t)=-\frac{1}{\tau_{\text{g}}}{\hat{b}}_{\text{g}}(t)$$

Taking the difference between real and estimated values, we obtain:

(7) $$\begin{array}{ll} \Delta \dot{\theta }(t) & =\dot{\theta }(t)-{\dot{\hat{\theta }}}_{\text{g}}(t)\\ & =b_{\text{g}}(t)-{\hat{b}}_{\text{g}}(t)+\eta_{b_{\text{g}}}(t)\\ & =\Delta b_{\text{g}}(t)+\eta_{b_{\text{g}}}(t) \end{array}$$

and:

(8) $$\begin{array}{ll} \Delta {\dot{b}}_{\text{g}}(t) & ={\dot{b}}_{\text{g}}(t)-{\dot{\hat{b}}}_{\text{g}}(t)\\ & =-\frac{1}{\tau_{\text{g}}}(b_{\text{g}}(t)-{\hat{b}}_{\text{g}}(t))+\eta_{b_{\text{g}}}(t)\\ & =-\frac{1}{\tau_{\text{g}}}\Delta b_{\text{g}}(t)+\eta_{b_{\text{g}}}(t) \end{array}$$

Hence, the state space representation of the estimation system is obtained as:

$$\begin{array}{ll} \dot{x} & =Ax(t)+Bw(t),\\ \left[\begin{array}{c} \Delta \dot{\theta }(t)\\ \Delta {\dot{b}}_{\text{g}}(t) \end{array}\right] & =\left[\begin{array}{rr} 0 & 1\\ 0 & -\frac{1}{\tau_{\text{g}}} \end{array}\right]\;\left[\begin{array}{c} \Delta \theta (t)\\ \Delta b_{\text{g}}(t) \end{array}\right]+\left[\begin{array}{cc} 1 & 0\\ 0 & 1 \end{array}\right]\;\left[\begin{array}{c} \eta_{\text{g}}(t)\\ \eta_{\text{b}}(t) \end{array}\right] \end{array}$$

where Δ*θ*(*t*) and Δ*b*_g_(*t*) are, respectively, the position estimation and offset errors of the gyroscope.

The angular position obtained from the accelerometer is given by:

(9) $${\hat{\theta }}_{a}(t)=\arcsin\left(\frac{a_{\text{a}}}{g_{e}}\right)=\theta (t)+\eta_{\text{a}}(t)$$

where *g_e_* is the gravitational acceleration constant. Here, the measured acceleration, *a*_a_, is assumed to contain only the component of the gravitational acceleration in the *x*-axis. That is, dynamic accelerations are not presented.

The output equation of the state space representation is given by the difference between the estimated position from the accelerometer and estimated position from the gyroscope, that is,

(10) $$\begin{array}{ll} z(t) & ={\hat{\theta }}_{\text{a}}(t)-{\hat{\theta }}_{\text{g}}(t)\\ & =\theta (t)+\eta_{\text{a}}(t)-{\hat{\theta }}_{\text{g}}(t)\\ & =\Delta \theta (t)+\eta_{\text{a}}(t) \end{array}$$

Therefore,

(11) $$z=Cx(t)+\upsilon (t)=\left[\begin{array}{cc} 1 & 0 \end{array}\right]\;\left[\begin{array}{c} \Delta \theta (t)\\ \Delta b_{\text{g}}(t) \end{array}\right]+\eta_{\text{a}}(t)$$

The final estimated values for the angular position and gyroscope bias are given by:

(12) $$\hat{\theta }(t)={\hat{\theta }}_{\text{g}}(t)+\hat{\Delta }\theta (t)$$

and:

(13) $${\hat{b}}_{\text{g}}(t)={\hat{b}}_{\text{g}}(t)+\hat{\Delta }b_{\text{g}}(t)$$

where Δ̂*θ* and Δ̂*b*_g_ are the estimated values of the angular position and offset errors, respectively. These errors are estimated by the Kalman filter using the sensor fusion approach.

### 2.3. Markovian Estimation Model

In this section, we propose a new MJLS model for angular position estimation of lower limb exoskeletons. Considering one leg of the exoskeleton, Exo-Kanguera (see Figures 1 and 2), the following absolute and relative angles are defined:

- Absolute angles: *θ_B_* (body/trunk), *θ_T_* (thigh), *θ_S_* (shank), *θ_F_* (foot);
- Relative angles: *θ_h_* (hip), *θ_k_* (knee), *θ_a_* (ankle).

The spatial configuration of the exoskeleton, Exo-Kanguera, at a given instant of time, can be obtained by estimating one absolute angle and three relative angles. The absolute angle is obtained by the sensor fusion approach presented in Section 2.2. The relative angles are measured by encoders coupled to the joints of the exoskeleton.

**Figure 3.**
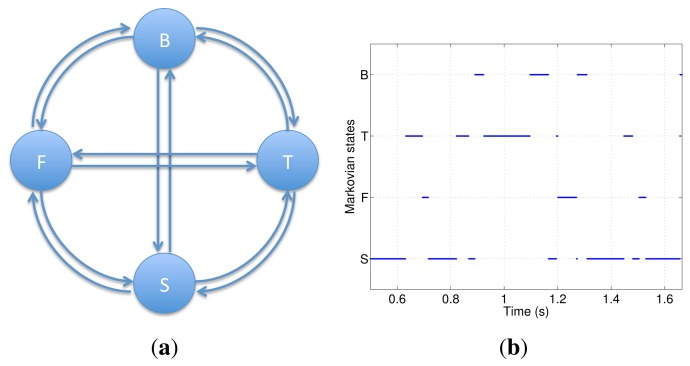
Markovian state jumps and model. (**a**) Markov Jump Linear Systems (MJLS) estimation model; (**b**) Markovian state jumps over one step.

Figure 3a shows the four Markovian states (B, Body; T, Thigh; S, Shank; F, Foot) of the proposed MJLS model. They are defined as the condition where the related IMU presents the smallest dynamic acceleration. In Figure 3b, we show the time intervals where each IMU reaches the Markovian states condition for one step. The incidence of dynamic accelerations is evaluated by computing the acceleration vector module, which includes all accelerometer axes. They are compared, in turn, to the gravitational acceleration.

The MJLS model for angular position estimation of lower limbs exoskeletons can be described by the following state-space equations:

(14) $$\dot{x}(t)=\bar{A}x(t)+\bar{B}w(t)$$

(15) $$y(t)=\bar{C}(\Theta (t))x(t)+\upsilon (t)$$

where *x*(*t*) ∈ ℝ*^n^* is the state vector, *w*(*t*) ∈ ℝ*^m^* and *υ*(*t*) ∈ ℝ*^q^* are, respectively, state and measurement noises, *y*(*t*) ∈ ℝ*^q^* is the vector output measurements and Θ(*t*) is the Markovian chain, with Θ(*t*) = {*B*, *T*, *S*, *F*}. The vectors of state and output measurements are defined, respectively, as:

(16) $$\begin{array}{ll} x & ={\left[\begin{array}{cccc} x_{B} & x_{T} & x_{S} & x_{F} \end{array}\right]}^{T}\\ & ={\left[\begin{array}{llllllll} \Delta \theta_{B} & \Delta b_{B} & \Delta \theta_{T} & \Delta b_{T} & \Delta \theta_{S} & \Delta b_{S} & \Delta \theta_{F} & \Delta b_{F} \end{array}\right]}^{T} \end{array}$$

(17) $$\begin{array}{ll} y & ={\left[\begin{array}{llll} \Delta_{\text{IMU}} & \Delta_{h} & \Delta_{k} & \Delta_{a} \end{array}\right]}^{T}\\ & ={\left[\begin{array}{llllllll} {\hat{\theta }}_{{\text{a}}_{\text{IMU}}} & -{\hat{\theta }}_{{\text{g}}_{\text{IMU}}} & \Delta \theta_{B} & -\Delta \theta_{T} & \Delta \theta_{T} & -\Delta \theta_{S} & \Delta \theta_{S} & -\Delta \theta_{F} \end{array}\right]}^{T} \end{array}$$

where Δ*θ_i_* = *θ_i_* − *θ̂*_g_*_i_*, for *i* = {*B*, *T*, *S*, *F*}, are the errors between the absolute angles (*θ_i_*) and the angle estimates calculated by the gyroscopes (*θ̂*_g_*_i_*); Δ*b_i_*, for *i* = {*B*, *T*, *S*, *F*}, are the errors of the bias generated by the gyroscopes for each segment, *i*; Δ*_IMU_* are the errors between the absolute angles estimates calculated for each segment by the accelerometers (*θ̂*_a*_IMU_*_) and the estimates calculated by the gyroscopes (*θ̂*_g*_IMU_*_); and Δ*_j_*, for *j* = {*h*, *k*, *a*}, are the errors of the relative angles related with the respective joints, *j*.

Considering the gyroscope, accelerometer and gyroscope bias models, matrices *Ᾱ* and *C̄* are defined as:

(18) $$\begin{array}{ll} \bar{A} & =\left[\begin{array}{cccccccc} 0 & 1 & 0 & 0 & 0 & 0 & 0 & 0\\ 0 & -\overset{1}{\;}/\underset{\tau_{{\text{g}}_{B}}}{\;} & 0 & 0 & 0 & 0 & 0 & 0\\ 0 & 0 & 0 & 1 & 0 & 0 & 0 & 0\\ 0 & 0 & 0 & -\overset{1}{\;}/\underset{\tau_{g_{T}}}{\;} & 0 & 0 & 0 & 0\\ 0 & 0 & 0 & 0 & 0 & 1 & 0 & 0\\ 0 & 0 & 0 & 0 & 0 & -\overset{1}{\;}/\underset{\tau_{g_{S}}}{\;} & 0 & 0\\ 0 & 0 & 0 & 0 & 0 & 0 & 0 & 1\\ 0 & 0 & 0 & 0 & 0 & 0 & 0 & -\overset{1}{\;}/\underset{\tau_{g_{F}}}{\;} \end{array}\right]\\ \bar{C}(\Theta (t)) & =\left[\begin{array}{cccccccc} M_{B}(t) & 0 & M_{T}(t) & 0 & M_{S}(t) & 0 & M_{F}(t) & 0\\ 1 & 0 & -1 & 0 & 0 & 0 & 0 & 0\\ 0 & 0 & 1 & 0 & -1 & 0 & 0 & 0\\ 0 & 0 & 0 & 0 & 1 & 0 & -1 & 0 \end{array}\right] \end{array}$$

where *M_i_*, for *i* = {*B*, *T*, *S*, *F*}, assumes values of zero or one, according to the angle associated with the value of Δ*_IMU_*. Thus, the only time-variant matrix is *C̄*(*t*). *B̄* is an identity matrix.

In the proposed approach, only one inertial sensor is used at each instant in combination with three relative sensors. As the system has four inertial sensors, the Markovian model presents four states, as shown in Figure 3a.

In Equation (18), four variables, *M_B_*(*t*), *M_T_*(*t*), *M_S_*(*t*) and *M_F_*(*t*), select the inertial sensor, which will be used at each time instant. Table 1 presents the Markovian states and the related variable values.

After generating the estimation errors for all angles from the Markovian filter, the estimated absolute angles are given by:

$$\begin{array}{ll} {\hat{\theta }}_{B} & ={\hat{\theta }}_{g_{B}}+\Delta {\hat{\theta }}_{B}\\ {\hat{\theta }}_{T} & ={\hat{\theta }}_{g_{T}}+\Delta {\hat{\theta }}_{T}\\ {\hat{\theta }}_{S} & ={\hat{\theta }}_{g_{S}}+\Delta {\hat{\theta }}_{S}\\ {\hat{\theta }}_{F} & ={\hat{\theta }}_{g_{F}}+\Delta {\hat{\theta }}_{F} \end{array}$$

## 3. Implementing

In this section, we present the Markovian Kalman filter (MKF) and the guidelines to choose the Markovian states for each instant of time. Consider the discrete-time version of the Markovian model, described in Section 2.3, given by:

(19) $$x_{k+1}=\Phi x_{k}+Gw_{k}$$

(20) $$z_{k}=Hx_{k}+\upsilon_{k}$$

where Φ = *I* + *ĀT*, *G* = *Ā*^−1^(Φ − *I*)*B̄* and *H*(*k*) = *C̄*. It is known from the literature that if (*z_k_*, *θ*(*k*)) are known, the best linear estimator of *x_k_*_+1_ is the Kalman filter (KF), since all operation modes are known at time *k* [11]. Therefore, the recursive predicting and updating equations for the MKF are given as follows

Predicting equations:
  (21) $${\hat{x}}_{k+1}=F_{k,\theta (k)}{\hat{x}}_{k|k}$$
  (22) $$P_{k+1}=F_{k,\theta (k)}P_{k|k}F_{k}^{T}+G_{k,\theta (k)}Q_{k}G_{k,\theta (k)}^{T}$$
Updating equations:
  (23) $$K_{k+1}=P_{k+1}H_{k+1,\theta (k)}^{T}{\left(H_{k+1,\theta (k)}P_{k+1}H_{k+1,\theta (k)}^{T}+R_{k+1}\right)}^{-1},$$
  (24) $${\hat{x}}_{k+1|k+1}={\hat{x}}_{k+1}+K_{k+1}(z_{k+1}-H_{k+1,\theta (k)}{\hat{x}}_{k+1}),$$
  (25) $$P_{k+1|k+1}=(I-K_{k+1}H_{k+1,\theta (k)})P_{k+1},$$
  where the weighting matrix, *Q*, is time-invariant and given by:
  (26) $$Q=\left[\begin{array}{cccccccc} \sigma_{{\text{g}}_{B}}^{2} & 0 & 0 & 0 & 0 & 0 & 0 & 0\\ 0 & \sigma_{b_{{\text{g}}_{B}}}^{2} & 0 & 0 & 0 & 0 & 0 & 0\\ 0 & 0 & \sigma_{{\text{g}}_{T}}^{2} & 0 & 0 & 0 & 0 & 0\\ 0 & 0 & 0 & \sigma_{b_{{\text{g}}_{T}}}^{2} & 0 & 0 & 0 & 0\\ 0 & 0 & 0 & 0 & \sigma_{{\text{g}}_{S}}^{2} & 0 & 0 & 0\\ 0 & 0 & 0 & 0 & 0 & \sigma_{b_{{\text{g}}_{S}}}^{2} & 0 & 0\\ 0 & 0 & 0 & 0 & 0 & 0 & \sigma_{{\text{g}}_{F}}^{2} & 0\\ 0 & 0 & 0 & 0 & 0 & 0 & 0 & \sigma_{b_{{\text{g}}_{F}}}^{2} \end{array}\right]$$

On the other hand, the weighting matrix, *R*, is considered time-variant, since it changes according to the Markov chain. It is given by:

(27) $$R=\left[\begin{array}{cccc} \sigma_{{\text{a}}_{\theta (k)}}^{2} & 0 & 0 & 0\\ 0 & \sigma_{e_{h}}^{2} & 0 & 0\\ 0 & 0 & \sigma_{e_{k}}^{2} & 0\\ 0 & 0 & 0 & \sigma_{e_{a}}^{2} \end{array}\right]$$

where $\sigma_{{\text{a}}_{\theta (k)}}^{2}$, for *θ*(*k*) = {*B*, *T*, *S*, *F*}, are given in Table 2.

### Required Conditions to Apply the Filter

The reliability of the accelerometer signal is essential to define the current Markovian state, in order to be used in the Kalman filter. When the module of the triaxial accelerometer vector is near the module of the gravity vector, there exists a low incidence of dynamic accelerations. We can define that the current incidence of dynamic accelerations and the Markovian state are chosen as:

(28) $$\rho (k)=\underset{i}{\min}(\left|\left\|a_{i}\right\|-g\right|)$$

(29) $$\theta (k)=\arg\underset{i}{\min}(\left|\left\|a_{i}\right\|-g\right|)$$

where *a_i_* are the values of each triaxial accelerometers, *θ*(*k*) = {*B*, *T*, *S*, *F*} describe each segment in lower limb exoskeletons, *θ*(*k*) is the current Markovian state and *ρ*(*k*) is an index that describes the reliability of the accelerometer used at the Markovian state, *θ*(*k*).

Thereby, the general condition to verify the reliability of the current IMU reading is given by:

(30) ρ(k)≤ζ

where *ζ* > 0 is a small value.

Let the magnitude vector measured by the four IMUs attached in a lower limbs exoskeleton (see Figure 2), for *k* = 1 to 10, be given by:

$$\begin{array}{ll} \left|\left\|a_{B}\right\|-g\right| & =(\begin{array}{cccccccccc} 0.8, & 0.9, & 0.6, & 0.5, & 0.4, & 0.1, & 0.2, & 0.4, & 0.3, & 0.1 \end{array})\\ \left|\left\|a_{T}\right\|-g\right| & =(\begin{array}{llllllllll} 0.7, & 0.7, & 0.4, & 0.2, & 0.6, & 0.2, & 0.2, & 0.3, & 0.6, & 0.2 \end{array})\\ \left|\left\|a_{F}\right\|-g\right| & =(\begin{array}{llllllllll} 0.3, & 0.4, & 0.3, & 0.3, & 0.3, & 0.4, & 0.3, & 0.4, & 0.9, & 0.8 \end{array})\\ \left|\left\|a_{S}\right\|-g\right| & =(\begin{array}{llllllllll} 0.1, & 0.2, & 0.7, & 0.6, & 0.5, & 0.5, & 0.1, & 0.5, & 0.1, & 0.7 \end{array}) \end{array}$$

Then, applying Equations (28) and (29), we obtain:

$$\begin{array}{ll} \rho (k) & =(\begin{array}{llllllllll} 0.1, & 0.2, & 0.3, & 0.2, & 0.3, & 0.1, & 0.1, & 0.3, & 0.1, & 0.1 \end{array})\\ \theta (k) & =(\begin{array}{llllllllll} S, & S, & F, & T, & F, & B, & S, & T, & S, & B \end{array}) \end{array}$$

if it is considered *ζ* = 0.2, through Equation (30), the Kalman filter will be updated for *k* = {1, 2, 4, 6, 7, 9, 10}.

Notice that we have two possible alternatives:

- Reliable measurement signal: In this case, the proposed MKF performs the prediction and update of the estimated angle, Equations (21) to (25).
- Unreliable measurement signal: In this case, the MKF performs only the prediction, Equations (21) and (22). The estimated angle is not corrected by the accelerometer measurement.

## 4. Results and Discussion

In order to present the simulation results, we consider the following assumptions:

- The angular estimate is performed for one leg of the exoskeleton (trunk to foot; see Figure 2).
- In the standard case, modeled in Section 2.2, the system is replicated for each segment of the exoskeleton. More details on the implementation of the standard Kalman filter can be seen in [8].
- In the Markovian case, modeled in Section 2.3, all segments are included in a unified model.
- For both estimate approaches, the weighting matrices parameters are given in Table 2. They were obtained based on optimization procedure given in [8].

**Figure 4.**
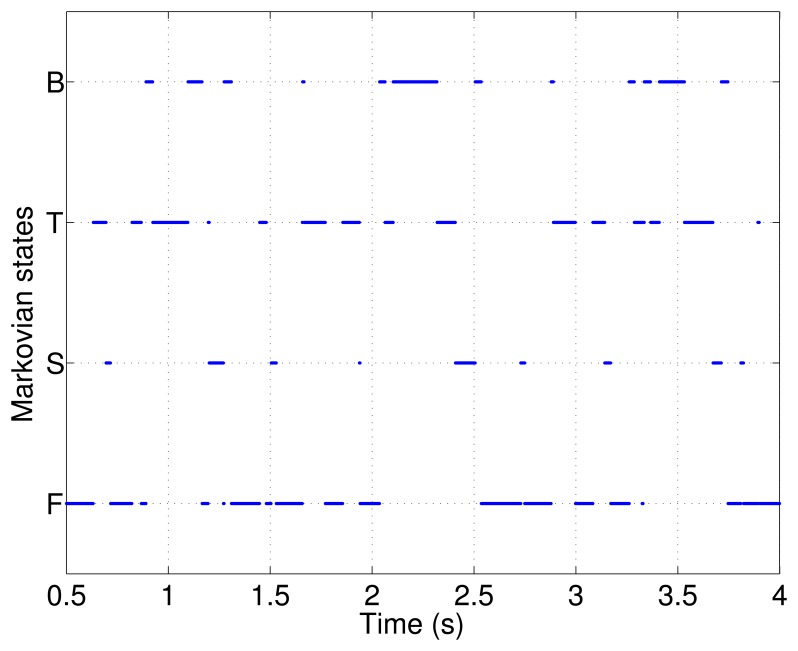
Markovian chain.

As discussed in Section 3, to define the sequence of Markov jumps among B, T, S and F, Equation (30) was used. The Markov chain was considered for three steps (see Figure 4).

**Figure 5.**
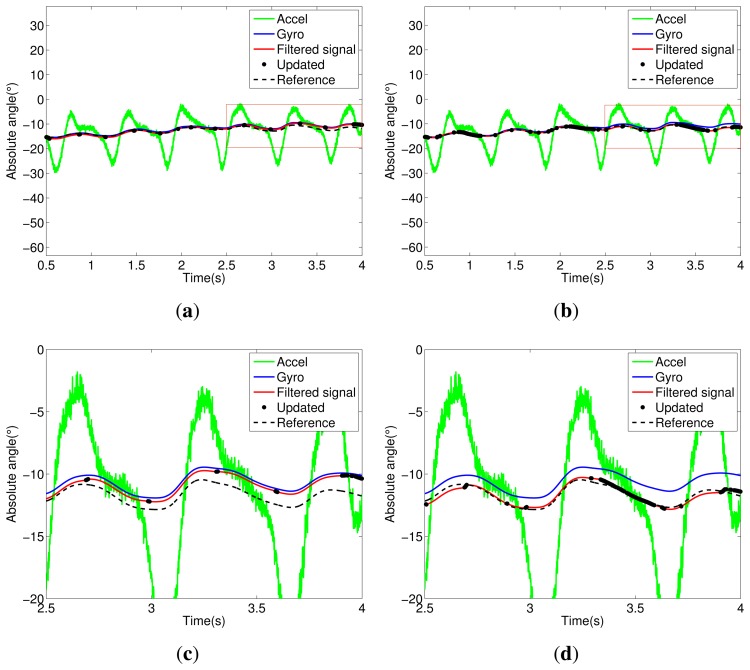
Body/trunk segment. (**a**) Kalman filter (KF) signals; (**b**) Markov KF (MKF) signals; (**c**) KF signals (zoom); (**d**) MKF signals (zoom).

**Figure 6.**
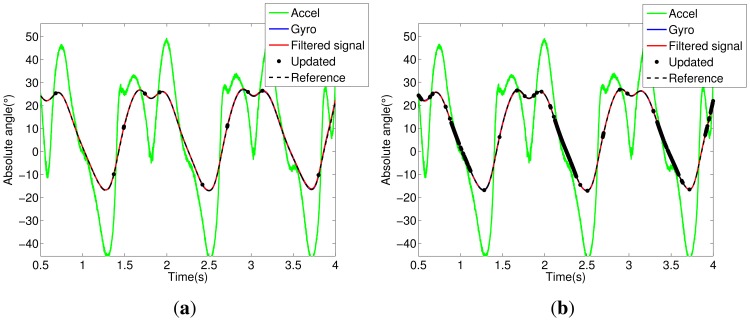
Thigh segment. (**a**) KF signals; (**b**) MKF signals.

**Figure 7.**
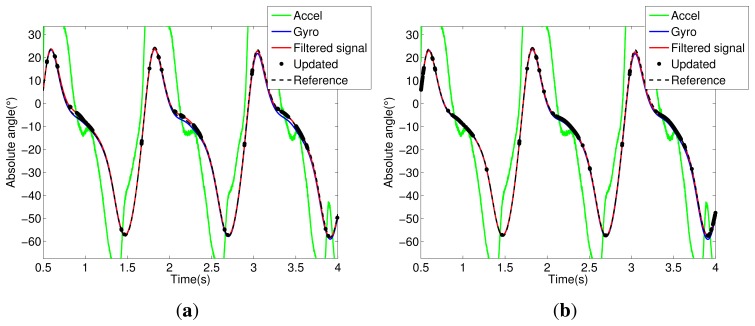
Shank segment. (**a**) KF signals; (**b**) MKF signals.

**Figure 8.**
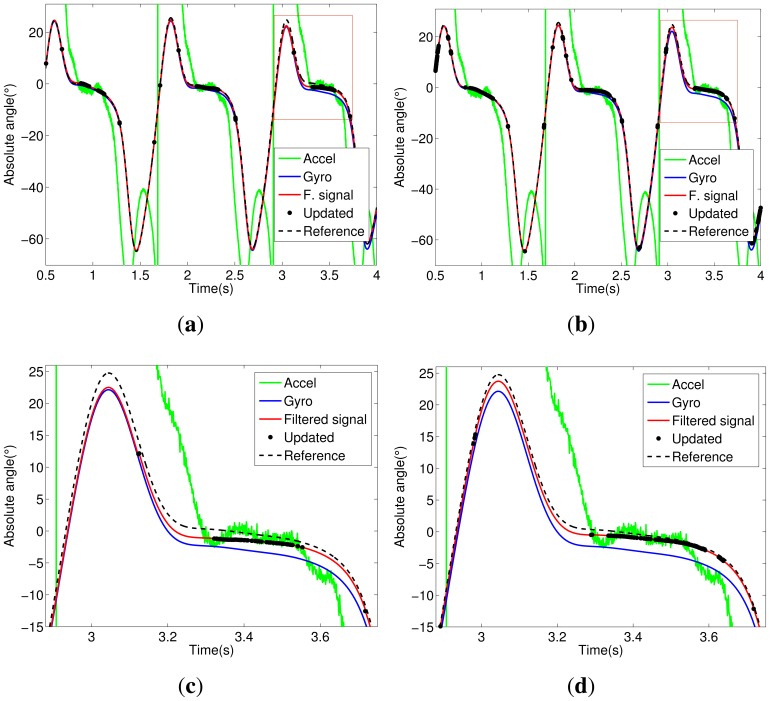
Foot segment. (**a**) KF signals; (**b**) MKF signals; (**c**) KF signals (zoom); (**d**) MKF signals (zoom).

Notice in Figure 5 that the trunk segment features have the most critical condition for angular estimation. It presents large dynamic acceleration levels cumulatively from the other segments (thigh, shank and foot) in all stages of walking. Thus, the accelerometer attached to the trunk has a reliable signal only for a few intervals; see the updated signals shown in Figure 5c. In Figure 5d, we can see the influence of the Markovian approach, which decreases the estimation error between filtered and reference signals. The same analysis can be extended to other segments of the exoskeleton, shown in Figures 5, 6, 7 and 8.

**Figure 9.**
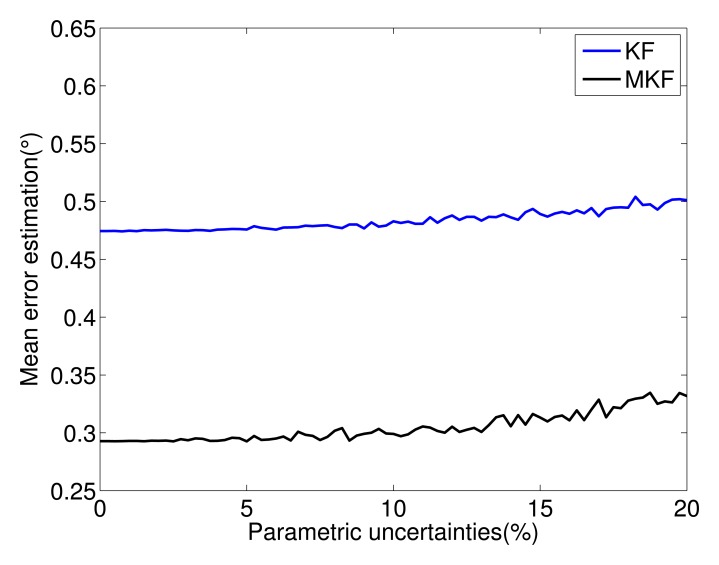
Filter effectiveness.

In order to verify the effectiveness of the filters when the system is subject to parametric uncertainties, both estimators were run, varying the components of the system matrices. Variations from 0% to 20% are assumed in the nominal values. Furthermore, 100 Monte Carlo simulations were performed. In Figure 9 are shown the estimation mean error for each filter. Notice the better performance of the Markovian approach over the standard Kalman filter for all ranges of uncertainties.

## 5. Conclusions

A MJLS-based position estimation approach for lower limbs exoskeletons is presented in this paper. Unlike the standard position estimations, where inertial sensors are designed to work individually for each link, the proposed approach considers a collective modeling of all inertial sensors attached to the exoskeleton. They are combined in a Markovian estimation model. Simulations results were presented considering the kinematic model of an exoskeleton for lower limbs, where four IMUs and three encoders were used. The results show the advantage of the proposed Markovian estimation model if compared with the standard one, even when they are subject to a wide range of parametric uncertainties.

## Figures and Tables

**Table 1.** Table of Markovian states.

**Modes of Operation (Θ(*t*))**	***M****_B_***(*t*)**	***M****_T_***(*t*)**	***M****_S_***(*t*)**	***M****_F_***(*t*)**
B	1	0	0	0
T	0	1	0	0
S	0	0	1	0
F	0	0	0	1

**Table 2.** System parameters.

**KF**	***ζ***	***σ***_g_	***σ****_b_*_g_	***σ***_a_	***τ***_g_
Body/Trunk	0.01	0.0103	0.0003	46.562	839.70
Thigh	0.01	0.0017	0.0005	329.08	2138.5
Shank	0.01	0.0008	9.6405	100.94	1465.3
Foot	0.01	7.7482	18.594	291.29	3567.5

## References

[1] Blaya J.B. (2002). Force Controllable Ankle-Foot Orthosis (AFO) to Assist Drop Foot Gait. Master's Thesis.

[2] Pons J., Moreno J., Brunetti F., Roccon E. (2007). Lower-Limb Wearable Exoskeleton.

[3] Frisoli A., Procopio C., Chisari C., Creatini I., Bonfiglio L., Bergamasco M., Rossi B., Carboncini M. (2012). Positive effects of robotic exoskeleton training of upper limb reaching movements after stroke. J. NeuroEng. Rehabil..

[4] Tong K., Granat M.H. (1999). A practical gait analysis system using gyroscopes. Med. Eng. Phys..

[5] Lau H., Tong K. (2008). The reliability of using accelerometer and gyroscope for gait event identification on persons with dropped foot. Gait Posture.

[6] Moreno J.C., de Lima E.R., Ruíz A.F., Brunetti F.J., Pons J.L. (2006). Design and implementation of an inertial measurement unit for control of artificial limbs: Application on leg orthoses. Sens. Actuators B Chem..

[7] Pappas I., Keller T., Mangold S., Popovic M., Dietz V., Morari M. (2004). A reliable gyroscope-based gait-phase detection sensor embedded in a shoe insole. IEEE Sens. J..

[8] Nogueira S.L., Inoue R.S., Terra M.H., Siqueira A.A.G. Estimation of Lower Limbs Angular Positions Using Kalman Filter and Genetic Algorithm.

[9] Nogueira S.L., Siqueira A.A.G., Inoue R.S., Terra M.H. Estimation of Lower Limbs Angular Positions Using Robst Kalman Filter and Genetic Algorithm.

[10] Siqueira A.A.G., Terra M.H. (2004). Nonlinear and Markovian 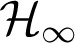
_∞_ controls of underactuated manipulators. IEEE Trans. Control Syst. Technol..

[11] Chizeck H., Ji Y. Optimal Quadratic Control of Jump Linear Systems with Gaussian Noise in Discrete-Time.

